# Imaging characteristics, yield of computed tomography, and clinical outcomes of central nervous system nocardiosis

**DOI:** 10.1007/s15010-026-02792-0

**Published:** 2026-04-14

**Authors:** Zachary A. Yetmar, Ryan B. Khodadadi, Supavit Chesdachai, Jack W. McHugh, Douglas W. Challener, Nancy L. Wengenack, Jason T. Little, Wendelyn Bosch, Maria Teresa Seville, Elena Beam

**Affiliations:** 1 Division of Public Health, Infectious Diseases, and Occupational Medicine, Mayo Clinic, Rochester, MN USA; 2 Department of Infectious Disease, Cleveland Clinic Foundation, 9500 Euclid Ave, G21, Cleveland, OH 44195 USA; 3 Division of Infectious Diseases, Vanderbilt University Medical Center, Nashville, TN USA; 4 Division of Clinical Microbiology, Mayo Clinic, Rochester, MN USA; 5 Department of Radiology, Mayo Clinic, Rochester, MN USA; 6 Division of Infectious Diseases, Mayo Clinic, Jacksonville, FL USA; 7 Division of Infectious Diseases, Mayo Clinic, Phoenix, AZ USA

**Keywords:** Brain abscess, CNS, CT, MRI, *Nocardia*

## Abstract

**Background:**

*Nocardia* frequently disseminates to the central nervous system (CNS). Few studies have described brain imaging characteristics of these infections or compared findings of computed tomography (CT) and magnetic resonance imaging (MRI).

**Methods:**

We conducted a retrospective cohort study of adults diagnosed with CNS nocardiosis between November 2011 and April 2022 and underwent brain MRI. We aimed to describe common brain imaging characteristics, compare the diagnostic yield of brain CT and MRI, and describe clinical outcomes.

**Results:**

52 patients were diagnosed with CNS nocardiosis, of which 26 also underwent brain CT. Most patients (N = 43; 82.7%) were receiving immunosuppressing medication, had concurrent pulmonary involvement (N = 45; 86.5%), and the most common species was *N. farcinica* (N = 19; 36.5%). 50% had CNS symptoms. The most common radiographic characteristics were supratentorial involvement (N = 50; 96.2%), multiple lesions (N = 31; 59.6%), and bihemispheric involvement (N = 30; 57.7%). Of 26 with both imaging modalities, 5 (19.2%) did not have signs of CNS nocardiosis on CT. These patients had smaller CNS lesions (median 0.6 versus 1.8 cm; *p* = 0.005) and more often had multiple brain lesions or bihemispheric involvement (both: 100% versus 47.6%; *p* = 0.053). Twelve (23.1%) patients died within 12 months of diagnosis, though abnormal CT and CNS lesion ≥ 1 cm were not associated with mortality. Of 33 who completed therapy, 3 (9.1%) and 2 (6.1%) patients had residual neurologic deficits or post-treatment recurrence, respectively.

**Conclusions:**

CNS nocardiosis commonly presents with multiple, supratentorial brain lesions. About 20% of patients will have a normal brain CT, usually those with multiple small lesions.

**Supplementary Information:**

The online version contains supplementary material available at 10.1007/s15010-026-02792-0.

## Background

*Nocardia* species are partially acid-fast saprophytic bacteria with a propensity for affecting a variety of distinct anatomic sites. While most originate from the lungs, up to 25% of *Nocardia* infections disseminate to the central nervous system (CNS) [1–3]. Patients who are immunocompromised tend to have higher rates of dissemination [3]. However, about half of immunocompromised patients with CNS nocardiosis have no clinical signs of CNS infection [4, 5]. Because of this, it is recommended that immunocompromised patients diagnosed with nocardiosis undergo brain imaging regardless of clinical symptoms to rule out CNS involvement [6–8].

While many patients with CNS nocardiosis undergo brain imaging, there are few studies defining common CNS imaging characteristics of this infection. Additionally, magnetic resonance imaging (MRI) is the most utilized brain imaging technique for CNS nocardiosis [9]. While this provides better resolution than computed tomography (CT), MRI is generally less accessible, more expensive, and contraindicated with certain medical devices. However, it is unclear whether CT provides adequate evaluation for CNS involvement. Therefore, we aimed to describe brain imaging among patients with CNS nocardiosis at our institution. Secondarily, we sought to compare radiologic characteristics of patients who underwent both CT and MRI brain imaging to estimate the rate of normal brain CT among patients with CNS nocardiosis and describe the rates of long-term outcomes.

## Methods

### Study design

We performed a multicenter, retrospective cohort study of adults with nocardiosis at three Mayo Clinic centers in Arizona, Florida, and Minnesota from November 2011 through April 2022. These data were originally collected as part of a previously published work [1]. Patients were ascertained from microbiology/culture records and screened through pre-determined criteria. Inclusion criteria included age ≥ 18 years, culture growth of a *Nocardia* species, and diagnosis of CNS nocardiosis. Exclusion criteria were lack of culture-confirmation of *Nocardia*, lack of brain MRI, and lack of research authorization per state statute. Nocardiosis was defined as culture growth of a *Nocardia* species with compatible signs, symptoms, and/or radiographic signs. CNS involvement was defined as growth of *Nocardia* species from a CNS specimen or radiographic evidence of CNS infection with culture-confirmation of *Nocardia* from another anatomic site. Once cases were screened for inclusion, data were manually extracted from the electronic medical record. Data included demographics, comorbid conditions, presenting characteristics, and radiographic characteristics. Radiographic characteristics were determined by a licensed neuroradiologist who specifically reviewed the CT and MRI studies for the purposes of this analysis. This review was performed in a blinded fashion without knowledge of the initial radiology interpretation, and the CT interpretation was performed independently of subsequent MRI review. We primarily aimed to describe CNS radiographic characteristics and rates of abnormal brain CT. Secondary outcomes included 1-year mortality, persistent neurologic deficits after 1 year, CNS imaging findings at the end of *Nocardia* therapy, and recurrent nocardiosis. Recurrent nocardiosis was defined as a new episode of nocardiosis occurring after completing treatment for the first episode. Study data were collected and managed using REDCap electronic data capture tools [10, 11]. This study was reviewed by our institutional review board and granted an exemption for informed consent (IRB #22–008346).

The clinical microbiology laboratory at Mayo Clinic in Rochester, Minnesota received specimens for culture, identification, and susceptibility testing from Mayo Clinic sites. Species identification and antimicrobial susceptibility testing was routinely attempted for all *Nocardia* isolates, and the utilized methods have been reported elsewhere [1].

### Statistical analysis

Continuous variables were summarized as median (interquartile range [IQR]) and categorical variables as number (percentage). Patients were categorized as having an abnormal or normal brain CT. Pertinent variables were compared between those with an abnormal or normal brain CT using Fisher’s exact test or Kruskal–Wallis rank sum test for categorical or continuous variables, respectively. Associations with 1-year mortality were assessed by unadjusted Cox regression. Due to the low number of events, Cox regression analyses were limited to pre-specified imaging characteristics. All analyses were performed using R version 4.3.1 (R Foundation for Statistical Computing, Vienna, Austria).

## Results

### Cohort characteristics

Among 374 patients with culture-confirmed nocardiosis, 53 had CNS involvement. One patient was excluded for only undergoing brain CT imaging, leaving 52 who underwent brain MRI and were included in this study. All patients had brain lesions on MRI. Of these 52, 26 (50%) underwent both CT and MRI. Most patients (N = 43; 82.7%) were receiving immunosuppressing medication, most commonly tacrolimus, mycophenolate, or prednisone. While 9 (17.3%) had chronic pulmonary disease, only 1 of these patients were not receiving immunosuppressing medication. Twenty-six (50%) had CNS symptoms at presentation. Most patients concurrently had pulmonary nocardiosis (N = 45; 86.5%), with a minority having cutaneous (N = 12; 23.1%) or bloodstream (N = 7; 13.5%) involvement. The most common *Nocardia* species were *N. farcinica* (N = 19; 36.5%) and *N. cyriacigeorgica* (N = 10; 19.2%; Supplementary Table 1). *Nocardia* susceptibility testing results were available for all isolates (Supplementary Table 2). Cohort characteristics are further detailed in Table 1.

**Table 1:** Baseline characteristics of 52 patients with central nervous system nocardiosis who underwent brain magnetic resonance imaging

	No CT (N = 26)	Underwent CT imaging (N = 26)	Total (N = 52)	*p*-value
Age, years	59.9 (55.9, 66.2)	66.4 (63.2, 69.7)	63.3 (57.3, 69.1)	0.034^1^
Sex				0.776^2^
Female	11 (42.3%)	9 (34.6%)	20 (38.5%)	
Male	15 (57.7%)	17 (65.4%)	32 (61.5%)	
Chronic pulmonary disease	3 (11.5%)	6 (23.1%)	9 (17.3%)	0.465^2^
Bronchiectasis	1 (33.3%)	0 (0.0%)	1 (11.1%)	0.226^2^
Chronic obstructive pulmonary disease	1 (33.3%)	5 (83.3%)	6 (66.7%)	
Interstitial lung disease	1 (33.3%)	1 (16.7%)	2 (22.2%)	
Solid organ transplant	17 (65.4%)	10 (38.5%)	27 (51.9%)	0.095^2^
Allogeneic stem cell transplant	3 (11.5%)	2 (7.7%)	5 (9.6%)	> 0.999^2^
Chronic lymphocytic leukemia	2 (7.7%)	2 (7.7%)	4 (7.7%)	> 0.999^2^
Immunosuppressant use	22 (84.6%)	21 (80.8%)	43 (82.7%)	> 0.999^2^
Tacrolimus	17 (65.4%)	11 (42.3%)	28 (53.8%)	0.164^2^
Mycophenolate	13 (50.0%)	10 (38.5%)	23 (44.2%)	0.577^2^
Corticosteroid	18 (69.2%)	18 (69.2%)	36 (69.2%)	> 0.999^2^
Other immunosuppression	3 (11.5%)	7 (26.9%)	10 (19.2%)	0.291^2^
Chronic pulmonary disease without immunosuppression	1 (3.8%)	0 (0.0%)	1 (1.9%)	> 0.999^2^
Non-CNS infection sites				
Pulmonary	23 (88.5%)	22 (84.6%)	45 (86.5%)	> 0.999^2^
Cutaneous	5 (19.2%)	7 (26.9%)	12 (23.1%)	0.743^2^
Bloodstream	5 (19.2%)	2 (7.7%)	7 (13.5%)	0.419^2^
Other site	6 (23.1%)	6 (23.1%)	12 (23.1%)	> 0.999^2^
Time from symptom onset to diagnosis, days	12.0 (9.0, 22.8)	14.5 (8.0, 35.2)	13.5 (8.0, 29.2)	0.985^1^
CNS symptoms	8 (30.8%)	18 (69.2%)	26 (50.0%)	0.012^2^
Headache	5 (19.2%)	7 (26.9%)	12 (23.1%)	0.743^2^
Confusion	2 (7.7%)	5 (19.2%)	7 (13.5%)	0.419^2^
Focal weakness	1 (3.8%)	12 (46.2%)	13 (25.0%)	< 0.001^2^
Paresthesia	1 (3.8%)	5 (19.2%)	6 (11.5%)	0.191^2^
*N. farcinica*	7 (26.9%)	12 (46.2%)	19 (36.5%)	0.249^2^
CNS imaging characteristics				
Multiple brain lesions	16 (61.5%)	15 (57.7%)	31 (59.6%)	> 0.999^2^
Bihemispheric involvement	15 (57.7%)	15 (57.7%)	30 (57.7%)	> 0.999^2^
Supratentorial involvement	24 (92.3%)	26 (100.0%)	50 (96.2%)	0.490^2^
Infratentorial involvement	9 (34.6%)	7 (26.9%)	16 (30.8%)	0.764^2^
Frontal lobe	17 (65.4%)	14 (53.8%)	31 (59.6%)	0.572^2^
Parietal lobe	14 (53.8%)	18 (69.2%)	32 (61.5%)	0.393^2^
Temporal lobe	8 (30.8%)	10 (38.5%)	18 (34.6%)	0.771^2^
Occipital lobe	12 (46.2%)	8 (30.8%)	20 (38.5%)	0.393^2^
Cerebellum	7 (26.9%)	6 (23.1%)	13 (25.0%)	> 0.999^2^
Diameter of largest CNS lesion, cm	0.7 (0.5, 1.0)	1.5 (0.8, 2.4)	1.0 (0.6, 1.8)	0.001^1^
Perilesional edema				0.004^1^
> 2 cm	6 (23.1%)	19 (73.1%)	25 (48.1%)	
1–2 cm	11 (42.3%)	4 (15.4%)	15 (28.8%)	
< 1 cm	6 (23.1%)	3 (11.5%)	9 (17.3%)	
None	3 (11.5%)	0 (0.0%)	3 (5.8%)	
Neurosurgical procedure	2 (7.7%)	12 (46.2%)	14 (26.9%)	0.004^2^
Craniotomy	1 (50.0%)	6 (50.0%)	7 (50.0%)	> 0.999^2^
Hemicraniectomy	0 (0.0%)	1 (8.3%)	1 (7.1%)	
Stereotactic needle biopsy	1 (50.0%)	5 (41.7%)	6 (42.9%)	
Neurosurgical indication				> 0.999^2^
Diagnostic	2 (100.0%)	9 (75.0%)	11 (78.6%)	
Therapeutic	0 (0.0%)	3 (25.0%)	3 (21.4%)	

CNS, central nervous system; CT, computed tomography. Data are median (interquartile range) or N (percentage) for continuous or categorical variables, respectively. ^1^Kruskal-Wallis rank sum test. ^2^Fisher’s Exact Test for Count Data

### CNS imaging characteristics

Among 52 patients, nearly all patients had at least one supratentorial lesion (N = 50; 96.2%). This most frequently involved the parietal lobe (N = 32; 61.5%) followed by the frontal (N = 31; 59.6%), occipital (N = 20; 38.5%), and temporal lobes (N = 18; 34.6%). Sixteen (30.8%) had an infratentorial lesion, most commonly involving the cerebellum (N = 13; 25.0%). The median diameter of the largest CNS lesion on MRI was 1.0 (IQR 0.6–1.8) cm, though most patients had multiple lesions (N = 31; 59.6%). Compared to other *Nocardia* species, patients with *N. farcinica* infection tended to have signs of nocardiosis on brain CT more often (91.7% versus 71.4%) and larger CNS lesions (median 1.5 versus 0.7 cm).

**Fig. 1:**
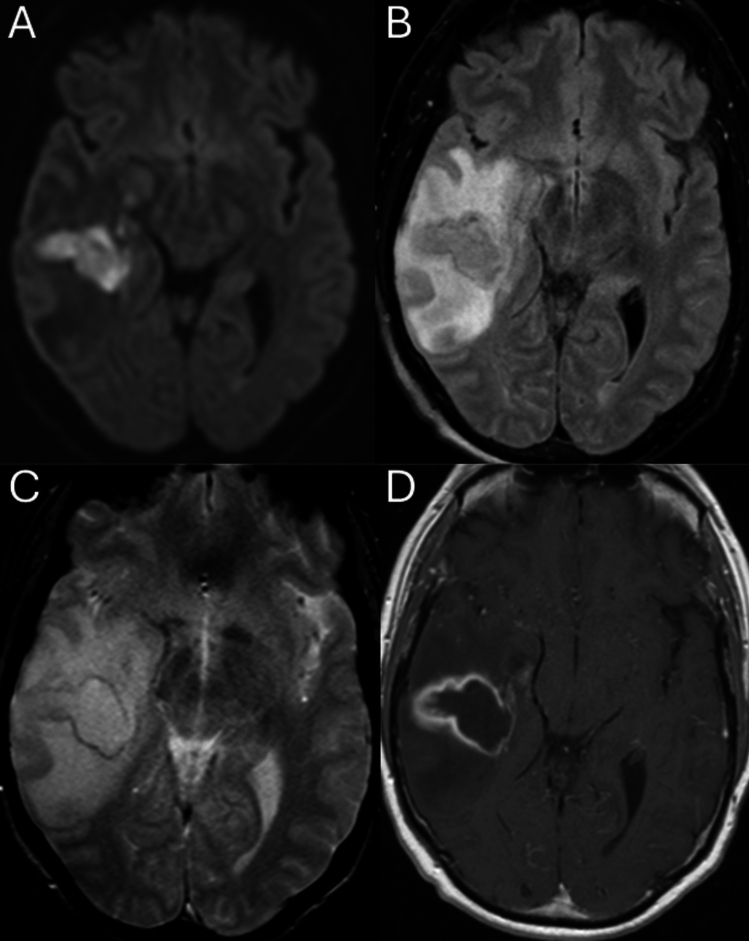
Magnetic resonance imaging of a patient with central nervous system nocardiosis. **A** Diffusion-weighted imaging shows marked internal diffusion restriction within the right temporal nocardia abscess. **B** FLAIR weighted images demonstrate marked perilesional edema. **C** Thin contiguous rim of hypointensity on gradient-recall echo. **D** Thick-walled peripheral enhancement of the abscess on postcontrast T1-weighted imaging, characteristic of a late capsule pyogenic abscess

Cerebral *Nocardia* infection in this study generally followed the well-described evolution of imaging appearance of pyogenic abscesses [12, 13]. Twenty-five (49.0%) patients had perilesional edema > 2 cm, 9 (17.3%) was 1–2 cm, 15 (28.8%) was < 1 cm, and 3 (5.8%) had no perilesional edema. Forty-seven (90.4%) had restricted diffusion on diffusion-weighted imaging. Forty-one (78.8%) patients had T2 hypointense or low gradient-recall echo (GRE) images. No patients had sulcal hyperintensity and only 3 (5.8%) patients had leptomeningeal enhancement. Patients were rarely imaged with MRI during the earliest stages of infection, including focal encephalitis without or with confluent central necrosis. Patients were much more frequently imaged during early capsulation of abscess, with coalescent core, faint diffusion restriction, and solid to moderately heterogeneous internal enhancement, or during late encapsulation, with thick enhancing wall and internal hypoenhancement and marked diffusion restriction [14]. Cerebral *Nocardia* abscesses with evidence of early to late encapsulation also frequently demonstrated peripheral linear hypointensity on susceptibility-weighted images and GRE images, previously described as characteristic of pyogenic brain abscess on MRI [15, 16]. Relative paucity of perilesional edema was noted in a minority of cases predominantly involving smaller abscesses, with moderate to marked edema associated with larger abscesses (Fig. 1).

### Comparison of brain CT and MRI

Among 26 patients who underwent imaging with both modalities, 5 (19.2%) had a normal brain CT (Table 2). Patients with a normal brain CT had a shorter time from symptom onset to *Nocardia* diagnosis (median 9 versus 17 days) and a lower rate of reported CNS symptoms (40.0% versus 76.2%). The CTs were performed a median of 1 day (IQR 0.0–2.0) before MRI. All patients with a normal head CT had multiple supratentorial lesions affecting both cerebral hemispheres, with a largest CNS lesion of 1.0 cm or smaller. This is contrasted by the 21 patients with abnormal brain CTs, who commonly had a single supratentorial brain lesion with a median diameter of 1.8 cm. Four (15.4%) CTs were with intravenous contrast, all of which had abnormal findings with the median largest CNS lesion size visualized being 2.0 cm (range 1.3–2.8 cm). Patients with abnormal CT were more likely to have perilesional edema > 2 cm (*p* < 0.001). Finally, all patients’ abnormal brain CT findings could not be radiographically distinguished from other etiologies with this pattern of a focal lesion with periphrenic enhancement, such as malignancy, prompting further characterization with MRI.

**Table 2:** MRI and procedural characteristics of 26 patients with central nervous system nocardiosis, comparing those with abnormal and normal head CT

	Abnormal head CT (N = 21)	Normal head CT (N = 5)	Total (N = 26)	*p*-value
Multiple brain lesions	10 (47.6%)	5 (100.0%)	15 (57.7%)	0.053^1^
Bihemispheric involvement	10 (47.6%)	5 (100.0%)	15 (57.7%)	0.053^1^
Supratentorial involvement	21 (100.0%)	5 (100.0%)	26 (100.0%)	
Infratentorial involvement	4 (19.0%)	3 (60.0%)	7 (26.9%)	0.101^1^
Brain lobes				
Frontal	11 (52.4%)	3 (60.0%)	14 (53.8%)	> 0.999^1^
Parietal	14 (66.7%)	4 (80.0%)	18 (69.2%)	> 0.999^1^
Temporal	7 (33.3%)	3 (60.0%)	10 (38.5%)	0.340^1^
Occipital	5 (23.8%)	3 (60.0%)	8 (30.8%)	0.281^1^
Cerebellum	4 (19.0%)	2 (40.0%)	6 (23.1%)	0.558^1^
Perilesional edema				< 0.001^1^
> 2 cm	19 (90.5%)	0 (0.0%)	19 (73.1%)	
1–2 cm	1 (4.8%)	2 (40.0%)	3 (15.4%)	
< 1 cm	1 (4.8%)	3 (60.0%)	4 (15.4%)	
Diameter of largest CNS lesion, cm				0.005^2^
Median (interquartile range)	1.8 (1.4, 2.5)	0.6 (0.5, 0.7)	1.5 (0.8, 2.4)	
Range	0.3—4.1	0.1—1.0	0.1—4.1	
Neurosurgical procedure	11 (52.4%)	1 (20.0%)	12 (46.2%)	0.330^1^
Craniotomy	5 (45.5%)	1 (100.0%)	6 (50.0%)	> 0.999^1^
Hemicraniectomy	1 (9.1%)	0 (0.0%)	1 (8.3%)	
Stereotactic needle biopsy	5 (45.5%)	0 (0.0%)	5 (41.7%)	
.				
Procedure indication				> 0.999^1^
Diagnostic	8 (72.7%)	1 (100.0%)	9 (75.0%)	
Therapeutic	3 (27.3%)	0 (0.0%)	3 (25.0%)	

Data are N (%) unless otherwise specified. *CNS* central nervous system, *CT* computed tomography. ^1^Fisher’s Exact Test for Count Data. ^2^Kruskal-Wallis rank sum test

### Treatment and outcomes

Twelve (23.1%) patients died within 1 year of *Nocardia* diagnosis, with a median time from diagnosis to death of 124.0 (IQR 41.8–225.0) days. Seven patients were lost to follow-up before 1-year, who had a median follow-up time of 137.0 (IQR 100.2–251.0) days. In total, 33 patients were alive with available follow-up 1 year after diagnosis. Three of 33 (9.1%) patients had residual neurologic deficits 1 year after starting treatment. Deficits included expressive aphasia, imbalance with vision deficits, and hemiparesis. Additionally, 31 of 33 with 1-year underwent follow-up CNS imaging via MRI prior to stopping therapy. This included 22 (71.0%) patients with hemosiderin, 21 (67.7%) with gliosis, 18 (58.1%) with both gliosis and hemosiderin, and 3 (9.7%) with encephalomalacia. Four (12.9%) patients had no follow-up abnormalities, or the only residual abnormalities were attributable to postoperative changes.

Patients who completed treatment received *Nocardia* therapy for a median of 365.0 (IQR 211.0–483.0) days. Eighteen (54.5%) patients received at least 1 year of therapy. This left 1 (3.0%) patient who received less than 120 days, 3 (9.1%) patients who received less than 180 days, 10 (30.3%) patients who received less than 240 days of treatment, 11 (33.3%) patients who received less than 300 days, and 15 (45.5%) patients who received less than 1 year of treatment. Treatment duration was similar between those who did and did not undergo neurosurgery (median 380.0 and 365.0 days, respectively). The most common initial antibiotics included trimethoprim-sulfamethoxazole (N = 35, 67.3%), imipenem (N = 23, 44.2%), linezolid (N = 21, 40.4%), and meropenem (N = 13, 25.0%). The median number of initial antibiotics was 2 (IQR 2–3), with 49 (94.2%) patients receiving at least 2 initial antibiotics (Supplementary Table 3).

**Fig. 2:**
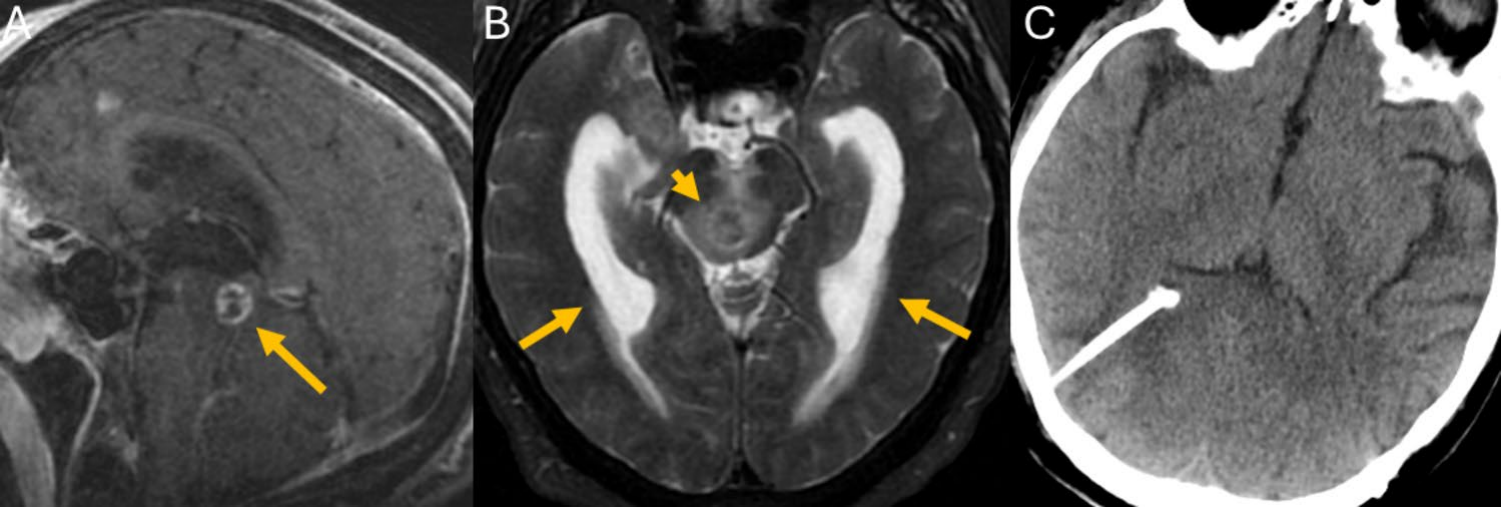
Magnetic resonance imaging of a patient with central nervous system nocardiosis who required neurosurgical intervention. **A** Sagittal postcontrast T1-weighted image shows a ring-enhancing abscess within the midbrain (arrow). **B** Axial T2-weighted image shows a hypointense nodular lesion, with perilesional edema in a periaqueductal region (short arrow), there is also obstruction of the cerebral aqueduct and enlargement of the lateral ventricles (long arrows), consistent with obstructive hydrocephalus. **C** Normalization of ventricle size on non-contrast computed tomography following placement of a ventricular shunt

Fourteen (26.9%) patients underwent neurosurgical intervention, most often for decompression of intracranial mass effect (Table 1). Others underwent needle biopsy and drainage due to failure to respond adequately to medical treatment, and to obtain cultures and antibiotic susceptibility testing. One patient underwent ventricular shunt placement to treat obstructive hydrocephalus secondary to mass effect from a midbrain abscess occluding the cerebral aqueduct (Fig. 2).

After completing treatment, 2 of 33 (6.1%) experienced *Nocardia* recurrence. Both recurrences were in the CNS with the same *Nocardia* species. One patient did not initially undergo neurosurgery, received 193 days of antibiotic therapy, and recurred 20 days after stopping treatment. The second patient did undergo initial neurosurgery, received 594 days of *Nocardia* therapy, and recurred 333 days after completing treatment. Both patients had follow-up CNS imaging at the end of therapy, each showing resolution of prior findings of nocardiosis.

In unadjusted Cox regression analyses, neither largest CNS lesion diameter ≥ 1 cm (hazard ratio [HR] 0.95, 95% confidence interval [CI] 0.33–3.21; *p* = 0.952) or abnormal brain CT (HR 1.09, 95% CI 0.12–9.75; *p* = 0.941) were associated with 1-year mortality. Compared to perilesional edema > 2 cm, perilesional edema of 1–2 cm (HR 1.27, 95% CI 0.23–6.93; *p* = 0.784) and < 1 cm (HR 1.93, 95% CI 0.52–7.20; *p* = 0.326) were not associated with mortality. Finally, presence of a single lesion compared to multiple lesions was not associated with mortality (HR 0.65, 95% CI 0.17–2.53; *p* = 0.539).

## Discussion

In this study, we characterize the CNS imaging findings of 52 patients with CNS nocardiosis. Supratentorial involvement was nearly universal, while a significant minority had infratentorial or cerebellar involvement. While multiple CNS lesions were common, only half of patients had CNS symptoms. Approximately 20% of patients with CNS nocardiosis who underwent brain imaging with CT and MRI had normal CT imaging despite clear abnormalities on MRI. However, neither a normal brain CT nor presence of a CNS lesion of 1 cm or more were associated with 1-year mortality.

Patients in this study commonly had extensive brain involvement from *Nocardia*, usually involving supratentorial structures. This is similar to prior studies, where patients commonly had multiple, large brain abscesses [17, 18]. Notably, compared to non-*Nocardia* brain abscesses, CNS nocardiosis is more commonly multifocal and involving infratentorial or posterior brain structures [19]. However, there is a wide range of brain imaging findings, and some patients did have solitary brain abscess or scattered, sub-centimeter lesions.

In the subset of patients with both imaging modalities, a significant proportion did not have signs of nocardiosis on CT. It stands to reason that brain MRI is the more sensitive imaging modality for CNS nocardiosis and should generally be favored. However, it is also important to consider the inherent differences between those with and without findings of nocardiosis on brain CT. Lack of abnormal signs on brain CT was associated with smaller CNS lesions, noting none of these patients had a CNS lesion with a diameter greater than 1.0 cm. Specifically, these patients also tended to have multifocal, bihemispheric lesions rather than one large, dominant abscess. Patients with multiple small lesions are less likely to undergo neurosurgical procedures. While data for CNS nocardiosis specifically is sparse, patients with bacterial brain abscess are more likely to require surgical drainage if they have a relatively large or solitary abscess [20]. This is difficult to assess in the present cohort due to the small sample size, though over half of those with an abnormal brain CT underwent a neurosurgical procedure compared to only 1 with a normal brain CT.

Imaging findings resemble those of pyogenic abscesses: on MRI, lesions show ring enhancement with gadolinium, a hypointense center on T1, hyperintensity on T2/FLAIR with a hypointense rim, and restricted diffusion in the core. They may present the "dual-rim sign" on susceptibility sequences. *Nocardia* abscesses are frequently multifocal, multilobulated, and less commonly associated with meningeal involvement [21].

Though patients with unrecognized CNS nocardiosis due to a falsely negative brain CT appear unlikely to require neurosurgery, antimicrobial choice and duration may be impacted with confirmation of CNS disease. However, while it is recommended that patients with CNS nocardiosis receive antimicrobials with adequate CNS penetration, most common *Nocardia* therapies, such as trimethoprim-sulfamethoxazole, linezolid, carbapenems, ceftriaxone, and fluoroquinolones, achieve acceptable drug concentrations in the CNS [22]. Thus, identification of small CNS lesions may not change management for most patients other than duration of total therapy, though the traditional treatment duration of 12 months for CNS nocardiosis is not evidence-based and patients have been successfully treated with shorter durations [23]. Additionally, we did not find evidence that patients without signs of nocardiosis on brain CT had worse mortality. Patients with brain lesions of at least 1 cm in diameter (an association with an abnormal brain CT) also did not have a differential effect on mortality. Similarly, about 45% of this cohort received less than 12 months of treatment with a rate of post-treatment recurrence similar to prior studies [24–26]. It is notable that recurrence developed in patients who received both short and prolonged therapy. It should also be noted that disseminated *Nocardia* infection has not been consistently shown to be associated with mortality in the modern era [1, 2, 26–28]. However, practitioners do commonly treat *Nocardia* lesions until radiographic resolution [6, 8], and brain imaging would not generally be repeated if CNS nocardiosis went unrecognized. Indeed, this practice was common in our cohort, though even patients with residual imaging abnormalities did not experience post-treatment recurrence. If follow-up imaging is assessed, it may be prudent to prioritize MRI given CT may miss subtle abnormalities and provide insufficient detail. However, our patients uniformly underwent follow-up MRI rather than CT, and this data does not provide a direct comparison in the follow-up setting.

This study has several limitations worth noting. It was retrospective and observational, and there are multiple sources of bias intrinsic to this study design. While large compared to some prior studies, we had relatively few patients which limited some analyses. Specifically, only half of the cohort had brain CT imaging, and differences between those who did and did not undergo CT imaging may have introduced spectrum bias which can affect generalizability. Most patients in this cohort were immunocompromised, and both radiographic findings and rates of negative brain CT may differ in immunocompetent populations. Additionally, most CTs were performed without intravenous contrast, and it is possible CTs with contrast have different performance. Not all patients from the original cohort underwent brain imaging, and there are likely some patients with unrecognized CNS nocardiosis who were excluded from this study. Finally, while we were able to report the rates of neurosurgical procedures, we were unable to analyze the effect of these or determine who may have benefited most from therapeutic CNS procedures.

In conclusion, most patients with CNS nocardiosis have multiple supratentorial brain lesions with 50% of patients endorsing CNS symptoms, highlighting that localizing symptoms are commonly absent despite CNS involvement in this largely immunocompromised cohort. About 20% with CNS nocardiosis lack signs of brain involvement on CT imaging. Patients with sub-centimeter lesions are more likely to have a normal brain CT. Brain CT screening may be reasonable to rule out a large CNS lesion, which would be more likely to require surgical intervention, though this may miss sub-centimeter lesions and MRI is typically required for further characterization even among those with an initial abnormal brain CT.

## Supplementary Information

Below is the link to the electronic supplementary material.

Supplementary file1 (DOCX 23 KB)

## Data Availability

Data from this analysis are available upon reasonable request to the corresponding author.

## Abbreviations

CI: Confidence interval
CNS: Central nervous system
CT: Computed tomography
GRE: Gradient-recall echo
HR: Hazard ratio
IQR: Interquartile range
MRI: Magnetic resonance imaging
