# Supplementary material for: Imaging characteristics, yield of computed tomography, and clinical outcomes of central nervous system nocardiosis
Source: Infection. 2026 Apr 14;54(3):1519–27. doi: 10.1007/s15010-026-02792-0 (PMC13193237; doi:10.1007/s15010-026-02792-0)
Supplement: Supplementary file 1 — Supplementary file1 (DOCX 23 KB) [file 15010_2026_2792_MOESM1_ESM.docx]

**Imaging Characteristics, Yield of Computed Tomography, and Clinical Outcomes of Central Nervous System Nocardiosis**

Zachary A. Yetmar^1,2^, Ryan B. Khodadadi^1,3^, Supavit Chesdachai^1^, Jack W. McHugh^1^, Douglas W. Challener^1^, Nancy L. Wengenack^4^, Jason T. Little^5^, Wendelyn Bosch^6^, Maria Teresa Seville^7^, Elena Beam^1^

^1^Division of Public Health, Infectious Diseases, and Occupational Medicine, Mayo Clinic, Rochester, Minnesota, USA

^2^Department of Infectious Disease, Cleveland Clinic Foundation, Cleveland, Ohio, USA

^3^Division of Infectious Diseases, Vanderbilt University Medical Center, Nashville, Tennessee, USA

^4^Division of Clinical Microbiology, Mayo Clinic, Rochester, Minnesota, USA

^5^Department of Radiology, Mayo Clinic, Rochester, Minnesota, USA

^6^Division of Infectious Diseases, Mayo Clinic, Jacksonville, Florida, USA

^7^Division of Infectious Diseases, Mayo Clinic, Phoenix, Arizona, USA

**Supplementary Table 1: *Nocardia* species**

|  | **Overall (N=52)** |
| --- | --- |
| ***N. abscessus*** | 3 (5.8%) |
| ***N. beijingensis*** | 1 (1.9%) |
| ***N. brasiliensis*** | 1 (1.9%) |
| ***N. cyriacigeorgica*** | 10 (19.2%) |
| ***N. farcinica*** | 19 (36.5%) |
| ***N. nova*** | 3 (5.8%) |
| ***N. otitidiscaviarum*** | 3 (5.8%) |
| ***N. paucivorans*** | 3 (5.8%) |
| ***N. pseudobrasiliensis*** | 2 (3.8%) |
| ***N. transvalensis*** | 1 (1.9%) |
| ***N. transvalensis/wallacei*** | 1 (1.9%) |
| ***N. veterana*** | 3 (5.8%) |
| ***N. wallacei*** | 2 (3.8%) |

**Supplementary Table 2: *Nocardia* antimicrobial susceptibility testing results**

|  | **Overall (N=52)** |
| --- | --- |
| **Amikacin** | 47 (90.4%) |
| **Amoxicillin-clavulanate** | 26 (50.0%) |
| **Cefepime** | 7 (13.5%) |
| **Ceftriaxone** | 19 (36.5%) |
| **Ciprofloxacin** | 17 (32.7%) |
| **Clarithromycin** | 12 (23.1%) |
| **Imipenem** | 35 (67.3%) |
| **Linezolid** | 52 (100.0%) |
| **Minocycline** | 16 (30.8%) |
| **Moxifloxacin** | 25 (48.1%) |
| **Tobramycin** | 20 (38.5%) |
| **Trimethoprim-sulfamethoxazole** | 48 (92.3%) |

Data are the number of isolates that tested susceptible to the specific agent.

**Supplementary Table 3: Initial antimicrobial treatment**

|  | **Overall (N=52)** |
| --- | --- |
| **Length of therapy, days** | 365.0 (211.0, 483.0) |
| **Amikacin** | 6 (11.5%) |
| **Cefepime** | 1 (1.9%) |
| **Ceftriaxone** | 7 (13.5%) |
| **Ciprofloxacin** | 2 (3.8%) |
| **Doxycycline** | 2 (3.8%) |
| **Imipenem** | 23 (44.2%) |
| **Linezolid** | 21 (40.4%) |
| **Minocycline** | 6 (11.5%) |
| **Moxifloxacin** | 5 (9.6%) |
| **Trimethoprim-sulfamethoxazole** | 35 (67.3%) |
| **Meropenem** | 13 (25.0%) |
| **Azithromycin** | 2 (3.8%) |
| **Sulfadiazine** | 4 (7.7%) |
| **Number of initial agents** | 2.0 (2.0, 3.0) |
| **Number of initial active agents^a^** | 2.0 (2.0, 3.0) |
| **≥2 initial agents** | 49 (94.2%) |
| **≥2 initial active agents^a^** | 45 (86.5%) |

Data are N (%) or median (interquartile range) for categorical and continuous variables, respectively.

^a^Active refers to antibiotics that tested susceptible from antibiotic susceptibility testing.
